# Terphenyllin Suppresses Orthotopic Pancreatic Tumor Growth and Prevents Metastasis in Mice

**DOI:** 10.3389/fphar.2020.00457

**Published:** 2020-04-08

**Authors:** Jia Zhang, Weiyi Wang, Yuan Zhou, Jing Yang, Jingli Xu, Zhiyuan Xu, Beihua Xu, Li Yan, Xiang-Dong Cheng, Minghua Li, Jiang-Jiang Qin

**Affiliations:** ^1^ Shanxi Province Academy of Traditional Chinese Medicine, Taiyuan, China; ^2^ College of Pharmaceutical Sciences, Zhejiang Chinese Medical University, Hangzhou, China; ^3^ Key Laboratory of Marine Biogenetic Resources, Third Institute of Oceanography, Ministry of Natural Resources, Xiamen, China; ^4^ First Clinical Medical College, Zhejiang Chinese Medical University, Hangzhou, China; ^5^ Institute of Cancer and Basic Medicine, Chinese Academy of Sciences, Hangzhou, China; ^6^ Cancer Hospital of the University of Chinese Academy of Sciences, Hangzhou, China; ^7^ Zhejiang Cancer Hospital, Hangzhou, China; ^8^ School of Pharmacy, Naval Medical University, Shanghai, China

**Keywords:** terphenyllin, pancreatic cancer, metastasis, Bax, Bcl-2, Puma

## Abstract

Pancreatic cancer (PC) is an aggressive and fatal disease with high incidences of metastasis and recurrence. However, there are no effective treatment options for the majority of PC patients, especially for those with locally advanced tumors and metastatic diseases. Therefore, it is urgently needed to develop safe and effective anti-PC therapeutic agents. We have recently identified a novel marine-derived natural product terphenyllin with potent anti-PC activity. The present study was designed to investigate the efficacy and mechanisms of action of terphenyllin in several human PC cell lines and an orthotopic PC mouse model. The results showed that terphenyllin significantly inhibited the viability of all PC cell lines with minimal effects on a normal human pancreatic cell line (HPNE). We next demonstrated the effects of terphenyllin on colony formation, apoptosis, migration, and invasion in both Panc1 and HPAC cell lines in a concentration-dependent manner. Terphenyllin also suppressed the tumor growth and metastasis in the Panc1 orthotopic mouse model. We further showed the profound effects of terphenyllin on the expression of apoptosis-associated proteins, including Bax, Bad, Puma, Bim_L_, Bcl-2, phos-Bcl-2 (Ser70), Bcl-xL, caspase 7, and PARP, which contributed to its anti-PC activity. In summary, terphenyllin suppressed the PC cell growth and metastasis *in vitro* and *in vivo* and may be developed as an anti-PC therapeutic agent in the future.

## Introduction

Pancreatic cancer (PC) is one of the most aggressive and fatal types of cancer and ranks the fourth leading cause of cancer-associated death worldwide with a dismal 5-year survival rate of 9% (Siegel et al., 2020). Despite the advancement in the development of new treatments, the therapeutic options for PC patients remain limited (Strobel et al., 2019). Surgical resection combined with chemotherapy provides PC patients with the only hope of long-term survival and cure (Kleeff et al., 2016). However, the majority of patients with early-stage PC are clinically silent, and only about 10% of patients are diagnosed at the resectable stage (Hartwig et al., 2013). Among the remaining 90% of PC patients, about 30% of them are diagnosed with locally advanced tumors and 60% even have metastatic diseases and poor performance status, which are generally recalcitrant to all forms of cancer treatment (Strobel et al., 2019). Therefore, there is an unmet need to develop novel therapies for improving the survival outcomes of PC patients as well as the quality of life.

Over the last few decades, chemotherapy with gemcitabine or 5-fluorouracil only demonstrates modest clinical benefit for PC patients (Burris et al., 1997; Kleeff et al., 2016). Chemotherapy combinations, such as FOLFIRINOX and gemcitabine plus nab-paclitaxel have significantly increased the overall survival of PC patients with advanced diseases (Mcbride et al., 2017; Nguyen et al., 2017). However, FOLFIRINOX has recently been associated with increased toxicity, mainly febrile neutropenia and diarrhea (Lambert et al., 2017). Numerous studies have unraveled the common molecular alterations occurring in PC, such as mutations in Kras, p53, and BRCA1 (Nag et al., 2013; Cicenas et al., 2017; Waters and Der, 2018), aberrant activation of wnt/β-catenin signaling and keap1/Nrf2 signaling (Qin et al., 2018; Kuo et al., 2019; Qin et al., 2019), and amplification and overexpression of MDM2, cyclin D1, USP7, and MDR1 (Qie and Diehl, 2016; Robey et al., 2018; Wang et al., 2019b; Dong et al., 2020; Qi et al., 2020), which play critical roles in the initiation, progression, metastasis, and chemoresistance of PC. Many targeted agents have been developed and evaluated in the preclinical and clinical settings (Karandish and Mallik, 2016). While several preclinical studies showed positive results (Wang et al., 2014b; Leal et al., 2017; Wang et al., 2018b), only a few of them, *e.g.* erlotinib have proved successful in PC clinical trials (Mosquera et al., 2016). Herein, safe and effective therapeutics are still urgently needed for PC therapy.

Natural products remain one of the most important sources for drug discovery and development (Qin et al., 2017a; Davison and Brimble, 2019). We have initiated an ongoing project aiming at identifying novel anticancer natural products from medicinal plants and marine-derived fungi and characterized several natural compounds with promising efficacy and safety profiles (Wang et al., 2012; Chen et al., 2013; Qin et al., 2015; Wang et al., 2018a). In a recent cancer cell-based screen, we have identified a cytotoxic natural product terphenyllin from a coral-derived fungus (Wang et al., 2017). Terphenyllin and its analogs have shown potent apoptosis-inducing ability in cancer cells (Wang et al., 2017; Wang et al., 2020). However, their *in vivo* efficacy and the molecular mechanisms are yet to be determined. The present study was designed to evaluate the anticancer efficacy of terphenyllin and its underlying mechanisms of action *in vitro* and *in vivo*. Our results demonstrate the therapeutic potential of terphenyllin in PC, which would provide a basis for further developing this natural compound as an anticancer therapeutic agent.

## Materials and Methods

### Cell Lines and Cell Culture

Human pancreatic cancer Panc1, HPAC, and SW1990 cell lines were obtained from the Cell Bank of the Chinese Academy of Science (Shanghai, China). The immortalized normal human pancreas cell line (HPNE) and human pancreatic cancer AsPC1 and CFPAC1 cell lines were kind gifts from Dr. Zhi-Gang Zhang (School of Medicine, Shanghai Jiao Tong University, Shanghai, China). The Panc1-Luc cell line was purchased from Meixuan Biological Science and Technology LTD (Shanghai, China). Panc1, SW1990, and HPNE cells were cultured in the DMEM medium. AsPC1 and CFPAC1 cells were maintained in the RPMI 1640 medium. HPAC cells were cultured in the DMEM/F12 (1:1) medium supplemented with 2 mg/L insulin, 5 mg/L transferrin, 40 ng/ml hydrocortisone, and 10 ng/ml epidermal growth factor. All media were supplemented with 10% fetal bovine serum (FBS) and 1% penicillin/streptomycin.

### Chemicals, Antibodies, and Other Reagents

**Figure 1:**
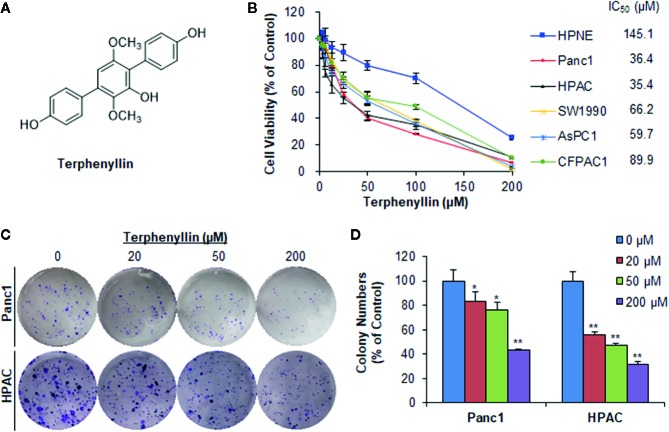
Terphenyllin inhibits pancreatic cancer cell viability and colony formation *in vitro*. **(A)** The chemical structure of terphenyllin. **(B)** HPNE, Panc1, HPAC, SW1990, AsPC1, and CFPAC1 cells were treated with terphenyllin at the indicated concentrations for 72 h, followed by CCK8 assays. **(C)** Panc1 and HPAC cells were treated with terphenyllin at the indicated concentrations for 24 h, followed by 10-day colony formation assays. **(D)** Quantitative analysis of colony formation. Data are representative of at least three experiments. (*p < 0.05, **p < 0.01).

The test compound terphenyllin (**Figure 1A**) was prepared in Dr. Weiyi Wang’s laboratory (Third Institute of Oceanography, Ministry of Natural Resources, Xiamen, China), and the structure was confirmed by NMR, MS, UV, and IR spectroscopy. The purity of terphenyllin was greater than 98%. All chemicals and solvents used were of the highest analytical grade available. The anti-rabbit Bax (D2E11), Bad (D24A9), Puma (D30C10), Bim (C34C5), Bcl-2 (D55G8), phos-Bcl-2 (p-Ser70) (5H2), Bcl-xl (54H6), caspase7 (D2Q3L), PARP (9542), and GAPDH (D16H11) antibodies were obtained from Cell Signaling Technology (Boston, USA). The goat anti-mouse IgG (H+L) and goat anti-rabbit IgG (H+L) antibodies were obtained from Bio-Rad (Hercules, CA, USA).

### Cell Viability Assay

The effects of terphenyllin on the cell viability were determined by Cell Counting Kit 8 (Nuoyang Biotech, Hangzhou, China). Briefly, HPNE, Panc1, HPAC, SW1990, AsPC1, and CFPAC1 cells were cultured in 96-well plates (2–3 × 10^3^ cells/well) overnight and then exposed to terphenyllin (3.125, 6.25, 12.5, 25, 50, 100, or 200 μM) or DMSO for 72 h. The treated cells were further incubated with CCK8 solution and the absorbance was measured at 450 nm by a Multiskan MK3 microplate reader (Thermo Scientific, USA). The cell viability and IC_50_ values were calculated as reported previously (Qin et al., 2016a).

### Colony Formation Assay

The colony formation assay was performed as described previously (Qin et al., 2017b; Wang et al., 2019a). Briefly, Panc1 and HPAC cells were seeded in 6-well plates (500 cells/well) overnight and treated with terphenyllin (20, 50, or 200 μM) or DMSO. After 24 h of exposure, the terphenyllin-containing medium was replaced with fresh medium without the test compound. The cells were grown for another 10 days, followed by fixation and crystal violet (Solarbio, China) staining.

### Apoptosis Assay

The effects of terphenyllin on cell apoptosis were performed as reported previously (Voruganti et al., 2015b). Briefly, 3 × 10^5^ Panc1 and HPAC cells in 6-well plates were exposed to terphenyllin (20, 50, or 200 μM) or DMSO for 48 h. The treated cells were harvested, washed with pre-cooling PBS, and then re-suspended in the mixture of binding buffer and staining reagents from FITC Annexin V Apoptosis Detection Kit I (BD Pharmingen, USA). The effects of terphenyllin on cell apoptosis were analyzed on a BD Accuri™ C6 flow cytometer (BD, Ann Arbor, MI, USA).

### Transwell Migration and Invasion Assays

The effects of terphenyllin on cell migration and invasion were determined using the transwell migration and invasion assays according to the manufacturers’ protocols (Wang et al., 2019a). For the migration assay, 5 × 10^4^ Panc1 and HPAC were suspended in 200 µl of serum-free medium, seeded in the upper compartment of the transwell chamber (Corning, USA), and incubated with terphenyllin (25 μM) or DMSO. Besides, 700 µl of complete medium with 20% FBS was added into the lower chamber. After 24 h of incubation, the cells on the upper surface of the membrane in the chambers were removed using cotton swabs whereas the cells migrated through the membrane were washed with PBS, stained with crystal violet (Solarbio, China), and analyzed under a microscope (Axio Observer A1, Zeiss, Germany). For the invasion assay, the upper surface of the membrane was covered with a layer of Matrigel (BD Biosciences, USA). The other procedures were similar to the migration assay.

### Western Blotting

Panc1 and HPAC cells were seeded in 6-cm dishes (3–5×10^5^ cells/well) overnight and exposed to terphenyllin (20, 50, or 200 μM) or DMSO for 24 h. The treated cells were then lysed with RIPA buffer (Absin Bioscience Inc, Shanghai, China) containing protease inhibitors (Solarbio Science & Technology Co., Ltd., Beijing, China) and phosphatase inhibitors (Roche, Switzerland). The cell lysates were centrifuged and the supernatants were collected, quantified, separated by an SDS-PAGE gel, and transferred to a PVDF blotting membrane (GE Healthcare, USA) for Western blot analysis following the manufacturer’s protocol (Xue et al., 2017; Qin et al., 2018). After blocking with 5% nonfat milk and incubation with primary and second antibodies, the blotting membranes were examined using ECL luminescence reagent (Absin, Shanghai, China), and the images were acquired on a FluorChem Q System (Alpha Innotech, Cell Bioscienes, USA).

### Panc1 Orthotopic Pancreatic Cancer Model

The orthotopic pancreatic cancer mouse model was developed as reported previously (Wang et al., 2014b; Wang et al., 2018b). Female 4–5-week-old SCID mice were purchased from the Shanghai Laboratory Animal Center (Shanghai, China). The experimental animal protocols were approved by the Board of Animal Study of Zhejiang Chinese Medical University. Briefly, 50 µl of Panc1-Luc cell solution (1 × 10^6^ cells in a 1:1 mixture of Matrigel and serum-free medium) was slowly injected into the head of the pancreas. Terphenyllin was dissolved in PEG400:ethanol:saline (57.1:14.3:28.6, v/v/v) and administered to mice by intraperitoneal injection at a dose of 20 mg/kg/day, 7 days/week for five weeks. For *in vivo* imaging, mice were administered intraperitoneally with fluorescein substrate (150 mg/kg) and anesthetized with isoflurane using an anesthesia machine (Summit Anesthesia, USA). The *in vivo* images for detecting the orthotopic tumor growth and metastasis were acquired on a Xenogen IVIS 200 imaging system (Caliper Life Sciences, USA). All the data analyses were performed using LT Living Image 4.3 Software. At the end of the experiments, all mice were examined for tumor metastasis to various organs.

### Hematoxylin and Eosin (H&E) Staining

The hematoxylin and eosin (H&E) staining was performed as described previously (Voruganti et al., 2015a; Qin et al., 2016b). At the termination of the *in vivo* experiments using the Panc1 orthotopic model, various tissues (liver, lungs, kidneys, spleen, heart, and brain) were removed from the tumor-bearing mice, fixed in 10% formalin, and embedded in paraffin. These tissue blocks were processed and sectioned at a thickness of 5 µm. The tissue sections were deparaffinized in xylenes, rehydrated, washed with PBS, stained in Mayer’s Hematoxylin for 10 min, and then stained with eosin for less than 1 min. After staining, the slides were dehydrated, mounted, and analyzed using an inverted microscope (Axio Observer A1, Zeiss, Germany).

### Statistical Analysis

All quantitative data were analyzed using the Prism software version 6 (Graph Pad Software Inc., San Diego, CA, USA) and are presented as means ± SEM derived from three or more independent experiments. The significance of differences for comparisons between two groups was analyzed using Student’s t-test. *p* < 0.05 was considered to be statistically significant.

## Results

### Terphenyllin Exerts Cytotoxicity in Human Pancreatic Cancer Cell Lines With Minimal Effects on Normal Pancreatic Cells

Terphenyllin (**Figure 1A**) was first tested for its cytotoxicity in five human PC cell lines (Panc1, HPAC, SW1990, AsPC1, and CFPAC1) and one normal human pancreatic cell line (HPNE) at various concentrations (0 to 200 μM) for 72 h. As shown in **Figure 1B**, terphenyllin significantly inhibited the growth of all the PC cell lines, with the IC_50_ values ranging from 35.4 to 89.9 μM. Among them, Panc1 and HPAC were the most sensitive cell lines with IC_50_ values of 36.4 and 35.4 μM, respectively. Of note, terphenyllin had minimal effects on the growth of the normal cell line HPNE, indicating the selective cytotoxicity of the compound against PC cells.

### Terphenyllin Inhibits the Colony Formation of Pancreatic Cancer Cells *In Vitro*

Terphenyllin was further evaluated for its *in vitro* anticancer activity in the most sensitive cell lines Panc1 and HPAC. As shown in **Figure 1C**, terphenyllin inhibited the cell colony formation in both cell lines in a concentration-dependent manner. At a high concentration of 200 μM, terphenyllin markedly decreased the numbers of colonies by 56.5% (*p* < 0.01) and 68.5% (*p* < 0.01) in Panc1 and HPAC cells, respectively (**Figure 1D**).

### Terphenyllin Induces Pancreatic Cancer Cell Apoptosis *In Vitro*

**Figure 2:**
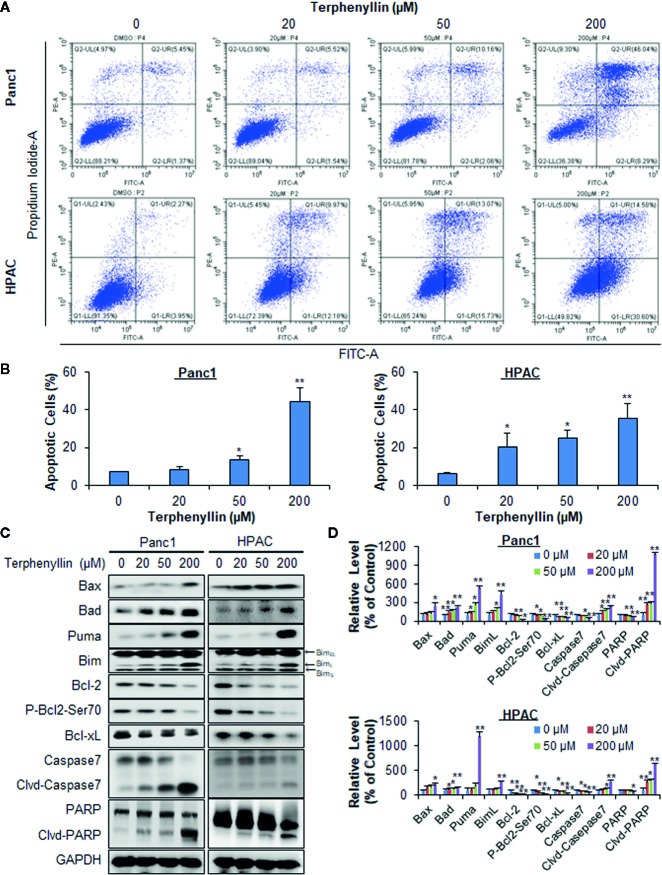
Terphenyllin induces the apoptosis of pancreatic cancer cells *in vitro*. **(A)** Panc1 and HPAC cells were treated with terphenyllin at the indicated concentrations for 48 h, followed by the detection of apoptosis by FITC-Annexin V assay. **(B)** The percentages of apoptotic cells. **(C)** Panc1 and HPAC cells were treated with terphenyllin at the indicated concentrations for 24 h, and the levels of various proteins were detected using specific antibodies by Western blotting analysis. **(D)** Relative band densities of various proteins. The densities of the protein bands were analyzed using ImageJ and normalized to GAPDH. Data are representative of at least three experiments. (*p < 0.05, **p < 0.01).

Terphenyllin was examined for its effects on cell apoptosis in Panc1 and HPAC cell lines. As shown in **Figure 2A**, terphenyllin induced significant apoptosis in both cell lines in a concentration-dependent manner. Panc1 cells were less sensitive to terphenyllin treatment than HPAC cells at lower concentrations (20 and 50 μM) but more sensitive at the higher concentration (200 μM). After exposure to 200 μM of terphenyllin for 48 h, 44.5% (*p* < 0.01) of Panc1 cells and 35.4% (*p* < 0.01) of HPAC cells underwent apoptosis, which were significantly higher than that of control cells (**Figure 2B**).

### Terphenyllin Modulates the Expression of Apoptosis-Related Proteins

To explore the mechanisms of action for the anticancer activity of terphenyllin, we examined its effects on the expression of key proteins involved in regulating cell apoptosis. As shown in **Figures 2C, D**, terphenyllin markedly increased the levels of pro-apoptotic proteins Bax, Bad, Puma, and Bim_L_ in both Panc1 and HPAC cell lines. The compound also decreased the levels of anti-apoptotic proteins Bcl-2 and Bcl-xL in both cell lines. It has been reported that the phosphorylation of Bcl-2 at Ser70 increases the anti-apoptotic activity of Bcl-2 by enhancing the dimerization with Bax (Deng et al., 2000; Deng et al., 2009). Terphenyllin reduced the expression of phosphorylated Bcl-2 (phos-Bcl-2) at Ser70, which may contribute to terphenyllin-induced apoptosis. The compound also cleaved and activated caspase 7 and PARP in both cell lines.

### Terphenyllin Suppresses Tumor Growth in an Orthotopic Pancreatic Cancer Model

**Figure 3:**
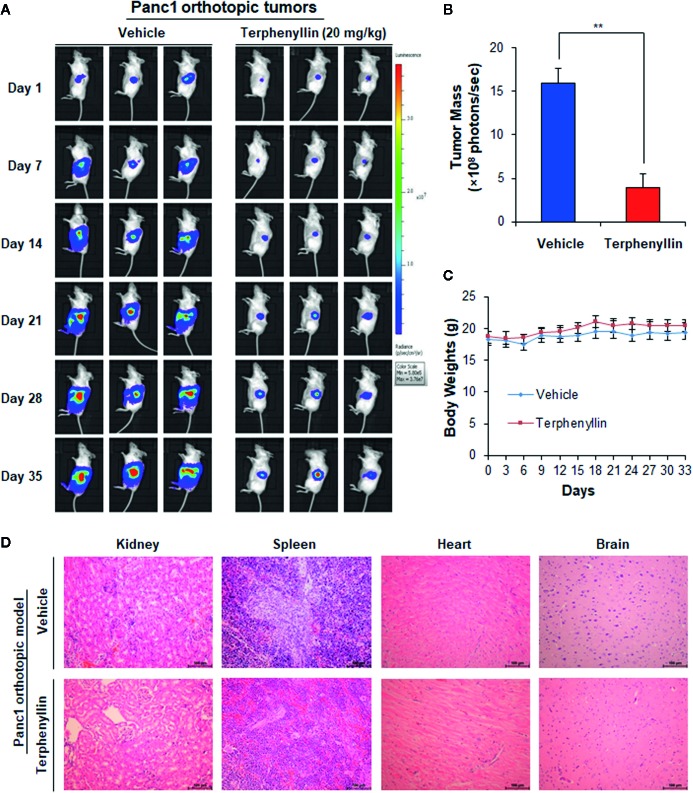
Terphenyllin suppresses the growth of Panc1 orthotopic tumors without causing any host toxicity. Panc1-Luc cells were implanted orthotopically into the pancreas of SCID mice. Mice were treated with terphenyllin by i.p. injection at doses of 20 mg/kg/d, 7 days/week for 5 weeks. **(A)** The luciferase signals in the mice bearing Panc1 orthotopic tumors were detected and images were obtained using an IVIS *in vivo* imaging system. **(B)** At the termination of the experiments, the average tumor mass (determined by the detected photons/sec) of the terphenyllin-treated mice was compared with that of the control mice. **(C)** The mice were monitored for changes in body weight as a surrogate marker for toxicity. **(D)** At the end of the experiments, the kidneys, spleen, heart, and brain were carefully removed from the mice bearing Panc1 orthotopic tumors, and H&E staining was performed on the paraffin sections of these tissues (all images represent serial sections; scale bar, 100 μm). (**p < 0.01).

We further assessed the *in vivo* efficacy of terphenyllin in the Panc1 orthotopic mouse model. As shown in **Figure 3A**, SCID mice bearing orthotopic Panc1 tumors were treated with vehicle or terphenyllin at 20 mg/kg/day, 7 days/week for five weeks, resulting in 75.5% inhibition of tumor growth compared with the vehicle-treated mice (**Figure 3B**). Importantly, terphenyllin treatment did not affect the average body weight of the mice in comparison to that of vehicle-treated mice, suggesting that the compound did not cause significant host toxicity during the treatment period (**Figure 3C**). At the end of the experiments, the kidneys, spleen, heart, and brain were carefully dissected from all mice for histological examinations. No abnormalities were observed in the organs from both vehicle- and terphenyllin-treated mice, indicating the absence of host toxicity (**Figure 3D**).

### Terphenyllin Prevents Pancreatic Cancer Cell Metastasis *In Vitro* and *In Vivo*

**Figure 4:**
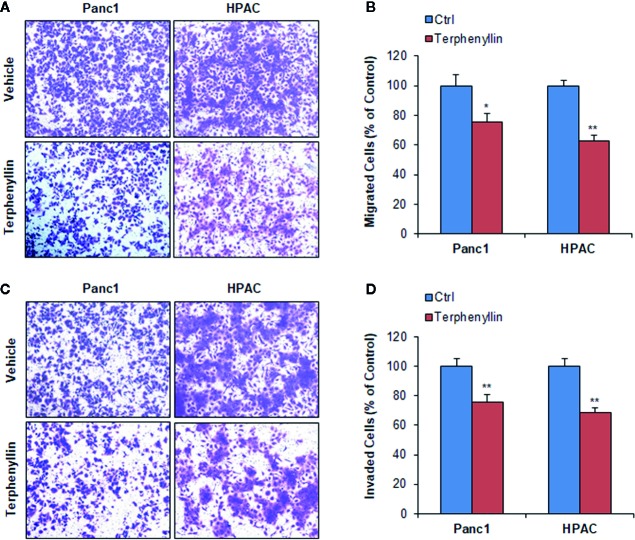
Terphenyllin inhibits pancreatic cancer cell migration and invasion *in vitro*. **(A)** Transwell migration assay was performed in Panc1 and HPAC cells with or without terphenyllin treatment. Representative microscopic images of cells that migrated through the transwell. **(B)** The quantitation of cells that migrated through the transwell in the migration assay. **(C)** Transwell Matrigel invasion assay was performed in Panc1 and HPAC cells with or without terphenyllin treatment. Representative microscopic images of cells that invaded through the transwell. **(D)** Quantitation of cells that invaded through the transwell in the Matrigel invasion assay. Data are representative of at least three experiments. (*p < 0.05, **p < 0.01).

**Figure 5:**
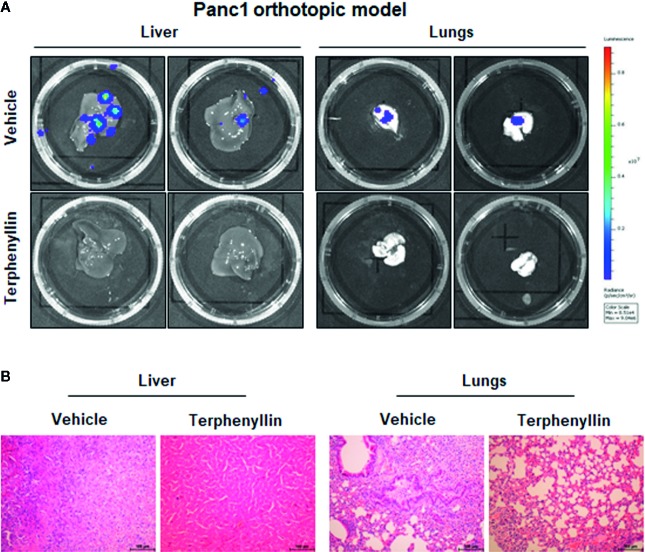
Terphenyllin prevents pancreatic tumor metastasis *in vivo*. **(A)** At the termination of the experiments using the Panc1 orthotopic model, the livers and lungs were carefully removed from the mice and imaged to detect metastatic lesions. **(B)** H&E staining was performed on the paraffin sections of these tissues (all images represent serial sections; scale bar, 100 μm).

We investigated the effects of terphenyllin on PC cell metastasis *in vitro*. As shown in **Figure 4A**, in a 24-h treatment period, terphenyllin at a sub-lethal concentration of 25 μM significantly reduced the migration of Panc1 and HPAC cells in the transwell migration assay by 24.3% (*p* < 0.05) and 37.0% (*p* < 0.01), respectively (**Figure 4B**). In the transwell Matrigel invasion assay, terphenyllin at 25 μM exerted similar preventive efficacy (**Figure 4C**) and decreased the numbers of invaded Panc1 and HPAC cells by 24.6% (*p* < 0.01) and 31.6% (*p* < 0.01), respectively (**Figure 4D**). At the end of the *in vivo* studies of the Panc1 orthotopic model, we examined the metastatic lesions in the liver and lungs from all mice (**Figure 5A**). The results showed that 5 and 4 out of 6 vehicle-treated mice developed metastatic lesions in the liver and lungs, respectively, whereas the incidence of liver and lung metastasis in terphenyllin-treated mice was decreased to 2/6 and 1/6, respectively. The histological examinations further confirmed the inhibition of liver and lung metastasis by the compound *in vivo* (**Figure 5B**).

## Discussion

Pancreatic cancer is a highly lethal and devastating disease with early metastasis and poor prognosis. Although the extensive molecular analyses of PC have indicated the strong genetic heterogeneity of this disease, some common molecular alterations have been characterized and validated as potential molecular targets for developing anti-PC therapeutic agents (Qie and Diehl, 2016; Cicenas et al., 2017; Waters and Der, 2018; Kuo et al., 2019; Qin et al., 2019). Despite the improvements in chemotherapy and targeted therapy and the considerable progress in increasing overall survival, there are very limited treatment options for PC patients, especially for those with locally advanced disease or metastasis (Werner et al., 2013; Rebelo et al., 2017; Neoptolemos et al., 2018).

Natural products provide a rich source of bioactive compounds with unique structures and diverse biological activities and play a crucial role in the discovery and development of anticancer therapeutics (Nag et al., 2012; Qian et al., 2013; Wang et al., 2014a; Kotecha et al., 2016; Kashyap et al., 2019). With a long-term goal of developing safe and effective anticancer agents, our laboratories have carried out cancer cell-based screenings of natural product libraries and identified several compounds with promising efficacy (Cheng et al., 2013; Li et al., 2013; Yao et al., 2017). The present study was designed to examine the anticancer efficacy of a novel marine-derived natural product terphenyllin and explore its molecular mechanisms in clinically relevant PC models *in vitro* and *in vivo*.

In the present study, we found that terphenyllin displayed significant cytotoxicity against human PC cell lines, while Panc1 and HPAC were the most sensitive cell lines. More importantly, we also observed that the normal HPNE cells were much less sensitive to the treatment of terphenyllin, suggesting the selective cytotoxicity toward cancer cells. Indeed, many natural products have potent cancer cell-killing properties; however, they also non-selectively kill normal cells, which largely limits their therapeutic value. In comparison to normal HPNE cells, terphenyllin displayed a selective growth inhibition of PC cancer cell lines, especially Panc1 and HPAC cell lines, which would be of great importance in the further development of this compound as an anticancer agent. In comparison to other PC cell lines, the selectivity indexes of terphenyllin against Panc1 and HPAC cells are relatively high. Therefore, we used Panc1 and HPAC as cell models for further evaluation of the compound. However, it should be noted that the selectivity index of terphenyllin against PC cell lines is still not good enough according to the “selectivity criteria”. Structural optimization of terphenyllin should be performed to improve its efficacy and selectivity in the future.

We further demonstrated that terphenyllin inhibited colony formation and induced apoptosis in PC cell lines in a concentration-dependent manner. To elucidate the molecular mechanisms for the anticancer activity of terphenyllin, we investigated its effects on apoptosis-related proteins. We found that terphenyllin induced PC cell apoptosis by increasing the expression levels of pro-apoptotic proteins (Bax, Bad, Puma, and Bim_L_) and decreasing the expression levels of anti-apoptotic proteins (Bcl-2 and Bcl-xL). We also observed that the compound reduced the expression of phos-Bcl-2 (Ser70) and cleaved and activated caspase 7 and PARP. However, this compound did not show any significant effects on several other members of the caspase family, including caspase 3 (data not shown). Its molecular target(s) and detailed mechanisms of action should be further investigated.

To confirm the anticancer efficacy of terphenyllin *in vivo*, we developed the Panc1 orthotopic mouse model, which could more closely mimic the original situation in human PC patients and better predict the therapeutic efficacy of the test compound. Our results showed the significant inhibitory effects of terphenyllin on tumor growth, as illustrated by *in vivo* imaging. Besides, the compound exhibited preventive effects on PC cell metastasis *in vitro*, as demonstrated by transwell migration and invasion assays. These preventive effects were further supported by the *in vivo* studies using the Panc1 orthotopic model. Of note, terphenyllin treatment did not cause any significant loss of mouse body weight or organ damage, indicating that the compound was safe at the effective dose. Nevertheless, it is necessary to examine the *in vivo* toxicity of terphenyllin by assessing the pathological sections of various organs from the terphenyllin-treated mice in our future studies. It has been demonstrated that treatment with different compounds may result in different toxicological responses; they may cause specific toxicity in a single organ (e.g., liver or lungs) or affect the entire body systemically (e.g., the immune system). Therefore, the examination of the organ toxicity will provide important information on the toxicological properties of terphenyllin, which is critically needed before we can move this compound into clinical trials. More clinically relevant PC models, such as patient-derived tumor models and transgenic mouse models are also expected for the further evaluation of terphenyllin.

In summary, the present study has shown that the marine-derived natural product terphenyllin suppresses PC tumor growth and metastasis *in vitro* and *in vivo* without causing significant toxicity at the effective dose. Although this study has demonstrated its efficacy, safety, and possible molecular mechanisms in PC cell lines *in vitro* and in the Panc1 orthotopic mouse model *in vivo*, future studies are warranted to determine its molecular targets, precise mechanisms of action, efficacy, and pharmacological and toxicological properties.

## Data Availability Statement

The datasets generated for this study are available on request to the corresponding authors.

## Ethics Statement

The animal study was reviewed and approved by the Board of Animal Study of Zhejiang Chinese Medical University.

## Author Contributions

JZ, WW, YZ, JY, JX, BX, and LY designed and conducted experiments, and wrote the manuscript. ZX and X-DC helped study design and interpretation of data. J-JQ and ML organized, conceived, and supervised the study. All authors read and approved the manuscript.

## Funding

This work was supported by National Natural Science Foundation of China (81903842, 81573953), Program of Zhejiang Provincial TCM Sci-tech Plan (2020ZZ005, 2016ZZ012), Zhejiang Chinese Medical University Startup Funding (111100E014), Medical Science and Technology Project of Zhejiang Province (WKJ-ZJ-1728), Traditional Chinese Medical Science and Technology Major Project of Zhejiang Province (2018ZY006), Science and Technology Projects of Zhejiang Province (2019C03049), Foundation of Third Institute of Oceanography SOA (2017001 and 2018021), Natural Science Foundation of Fujian Province (2018J01064), and COMRA program (DY135-B2-05 and DY135-B2-01).

## Conflict of Interest

The authors declare that the research was conducted in the absence of any commercial or financial relationships that could be construed as a potential conflict of interest.
