# Supplementary material for: Terphenyllin Suppresses Orthotopic Pancreatic Tumor Growth and Prevents Metastasis in Mice
Source: Front Pharmacol. 2020 Apr 8;11:457. doi: 10.3389/fphar.2020.00457 (PMC7157903; doi:10.3389/fphar.2020.00457)

Supplementary Information

Terphenyllin Suppresses Orthotopic Pancreatic Tumor Growth and Prevents Metastasis in Mice

Jia Zhang[1,2†], Weiyi Wang[3†*], Yuan Zhou[2†], Jing Yang[2†], Jingli Xu[4], Zhiyuan Xu[5], Beihua Xu[2], Li Yan[6], Xiang-Dong Cheng[5], Minghua Li[1*], Jiang-Jiang Qin[2,5*]

[1]Shanxi Province Academy of Traditional Chinese Medicine, Taiyuan 030012, China
[2]College of Pharmaceutical Sciences, Zhejiang Chinese Medical University, Hangzhou 310053, China
[3]Key Laboratory of Marine Biogenetic Resources, Third Institute of Oceanography, Ministry of Natural Resources, Xiamen 361005, China
[4]First Clinical Medical College, Zhejiang Chinese Medical University, Hangzhou 310053, China
[5]Institute of Cancer and Basic Medicine, Chinese Academy of Sciences; Cancer Hospital of the University of Chinese Academy of Sciences; Zhejiang Cancer Hospital, Hangzhou 310022, China
[6]School of Pharmacy, Naval Medical University, Shanghai 200433, China

[†]These authors have contributed equally to this work.

*** Correspondence:**
Jiang-Jiang Qin
jqin@zcmu.edu.cn or zylysjtu@hotmail.com

Weiyi Wang
wywang@tio.org.cn

Minghua Li
1035448060@qq.com

Supplementary Figures

Supplementary Figure 1. Original images of anti-Bax immunoblots in Figure 2C.

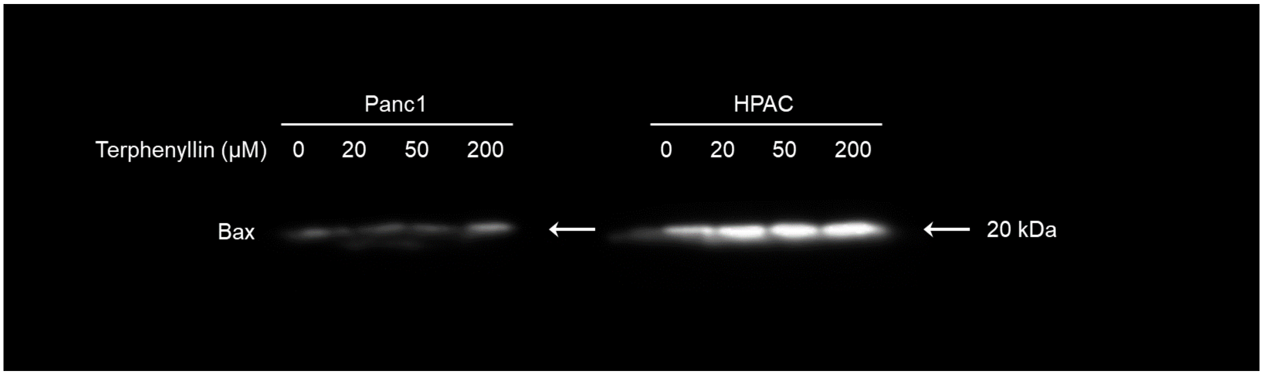

Supplementary Figure 2. Original images of anti-Bad immunoblots in Figure 2C.

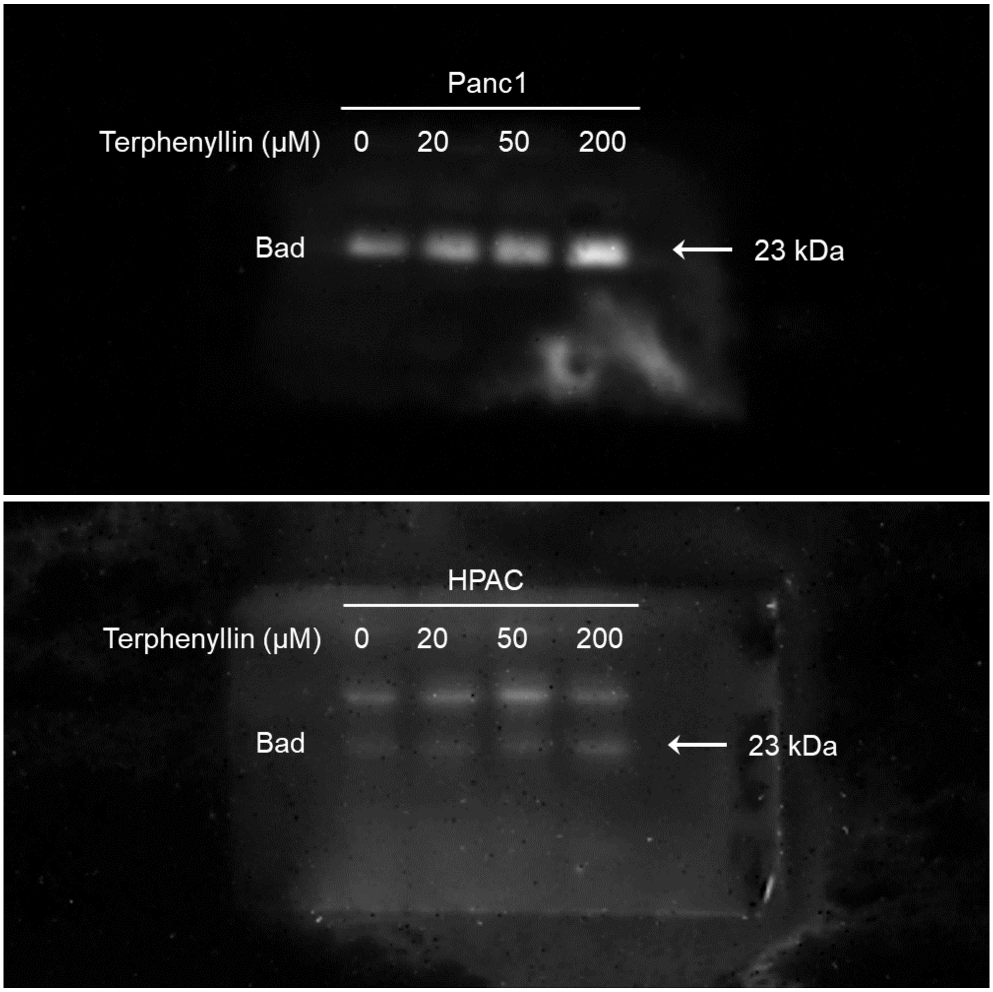

Supplementary Figure 3. Original images of anti-Puma immunoblots in Figure 2C.

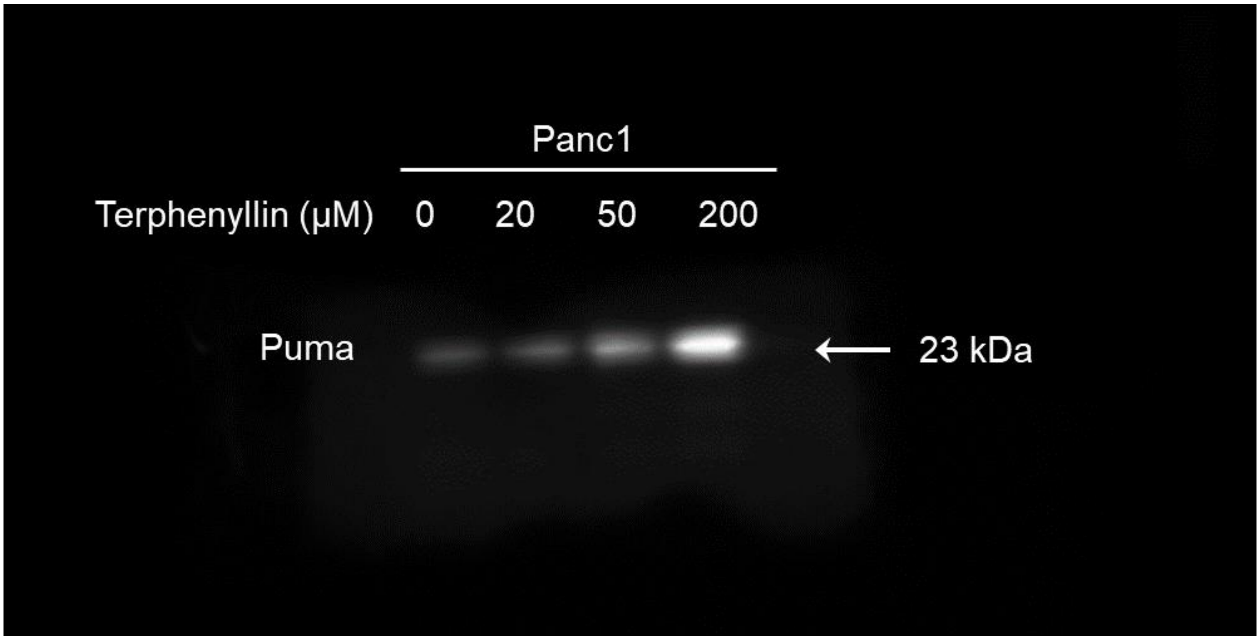

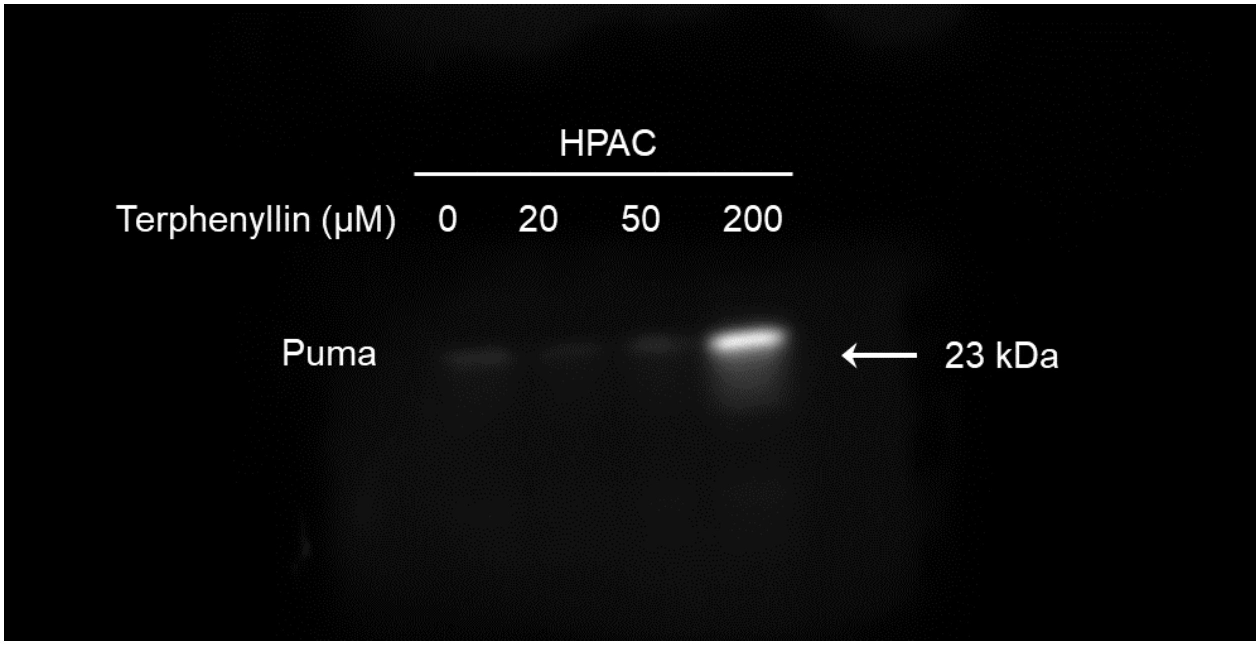

Supplementary Figure 4. Original images of anti-Bim immunoblots in Figure 2C.

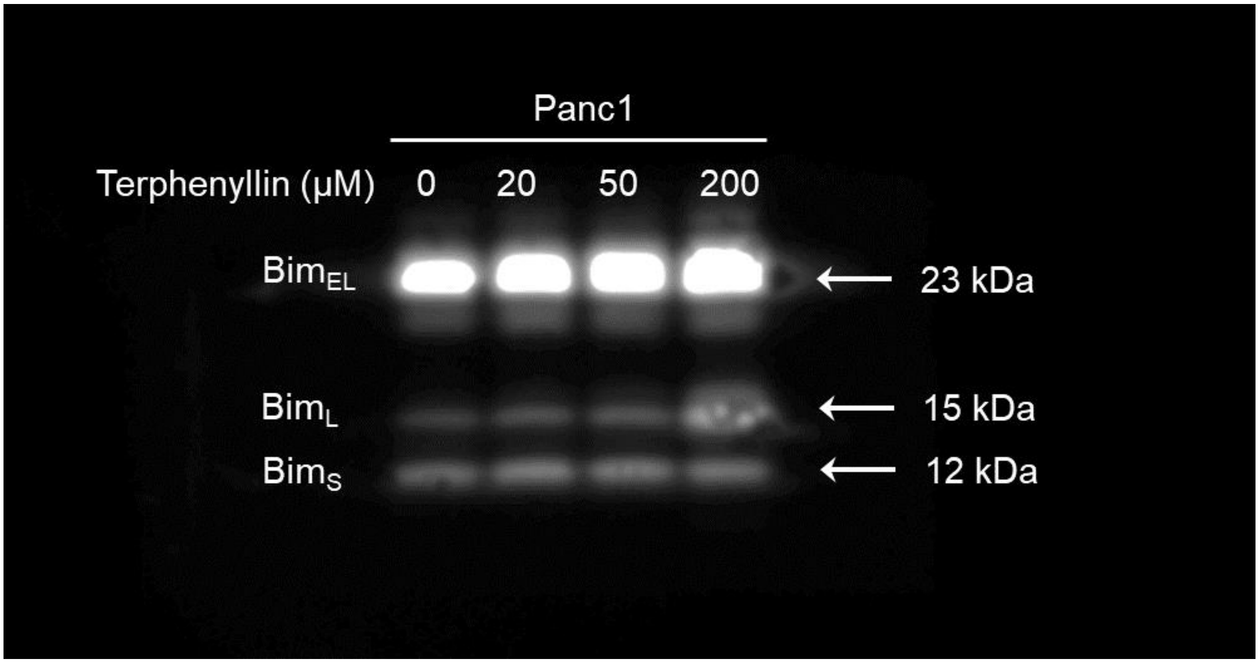

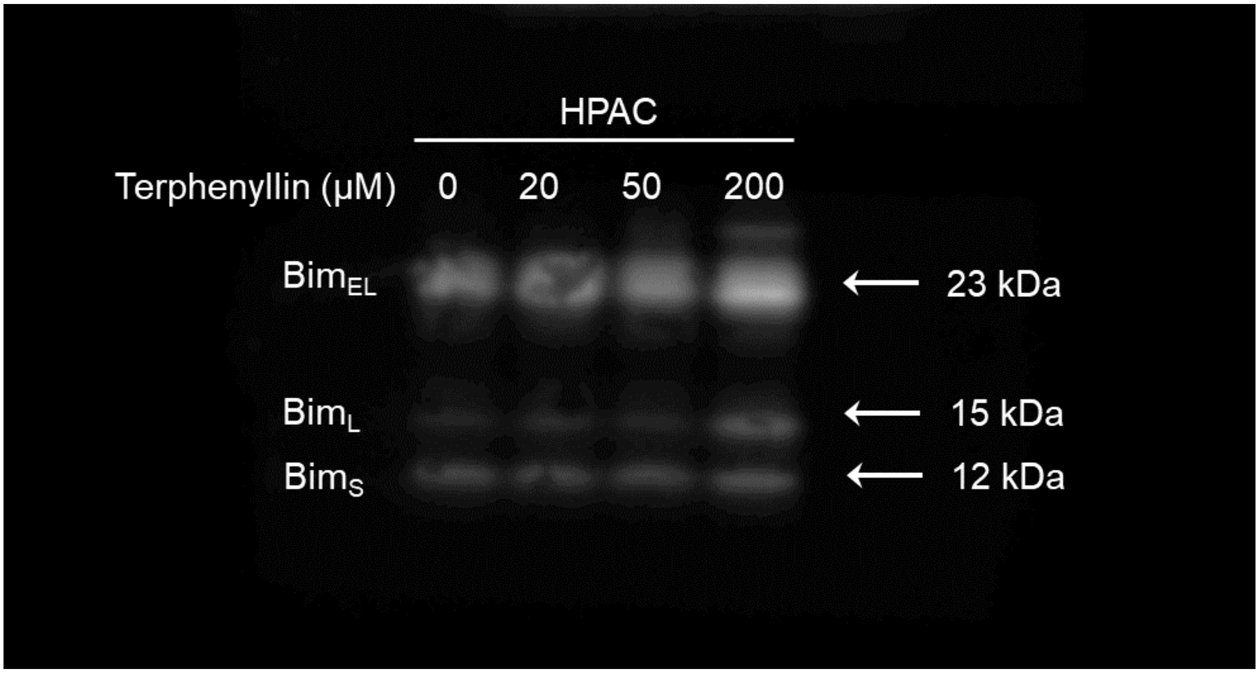

Supplementary Figure 5. Original images of anti-Bcl-2 immunoblots in Figure 2C.

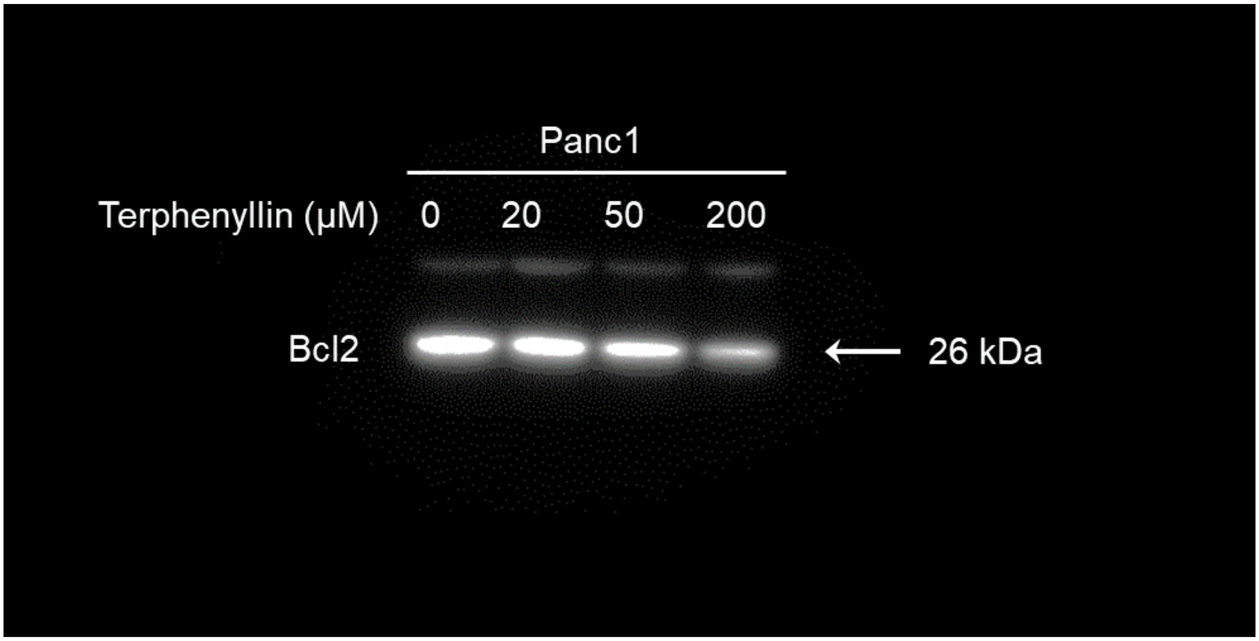

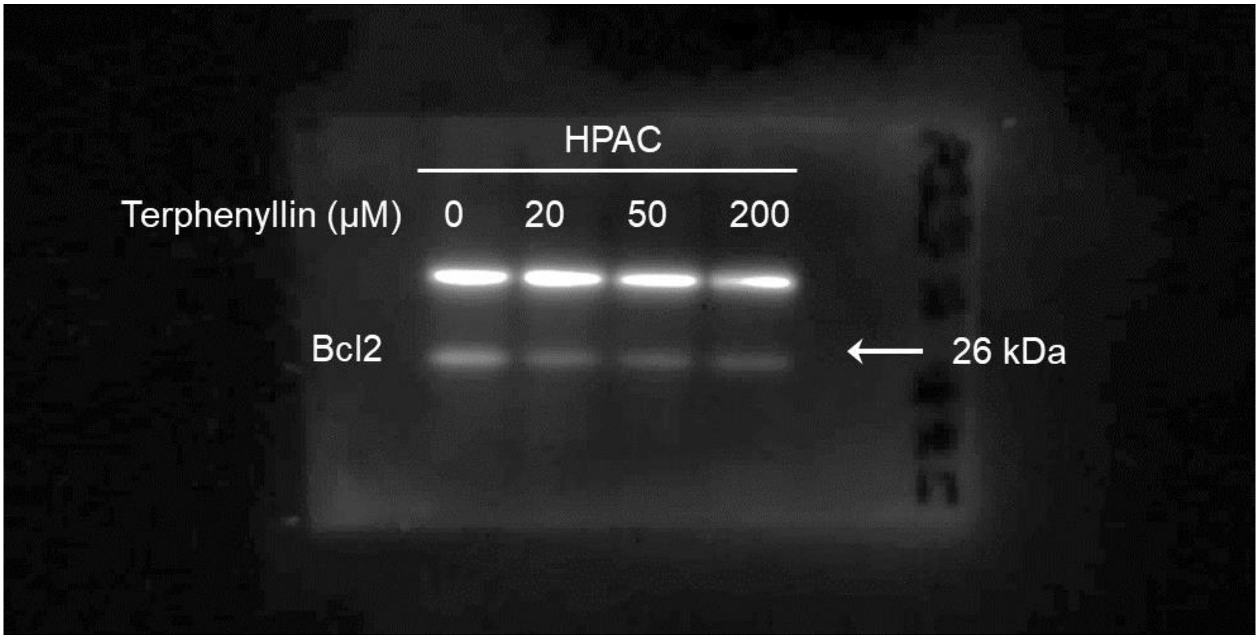

Supplementary Figure 6. Original images of anti-p-Bcl-2-Ser70 immunoblots in Figure 2C.

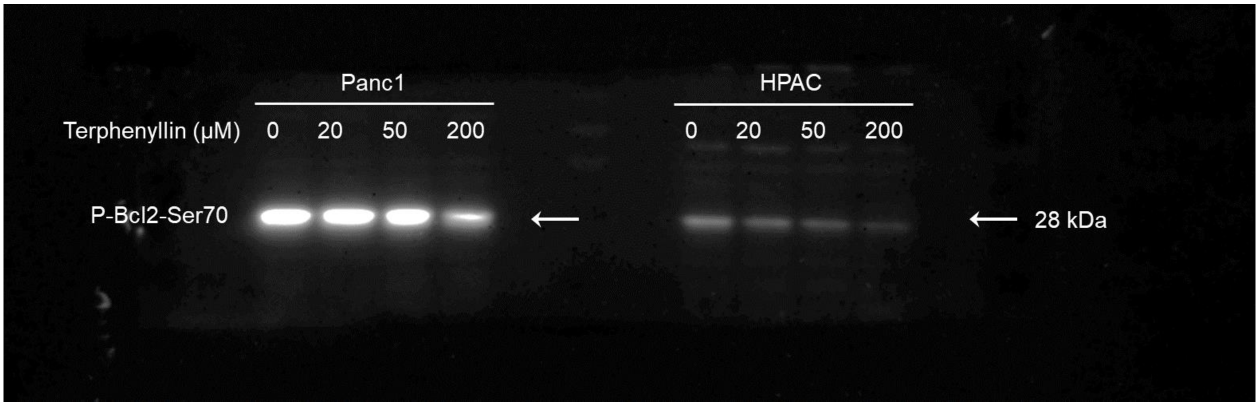

Supplementary Figure 7. Original images of anti-Bcl-xL immunoblots in Figure 2C.

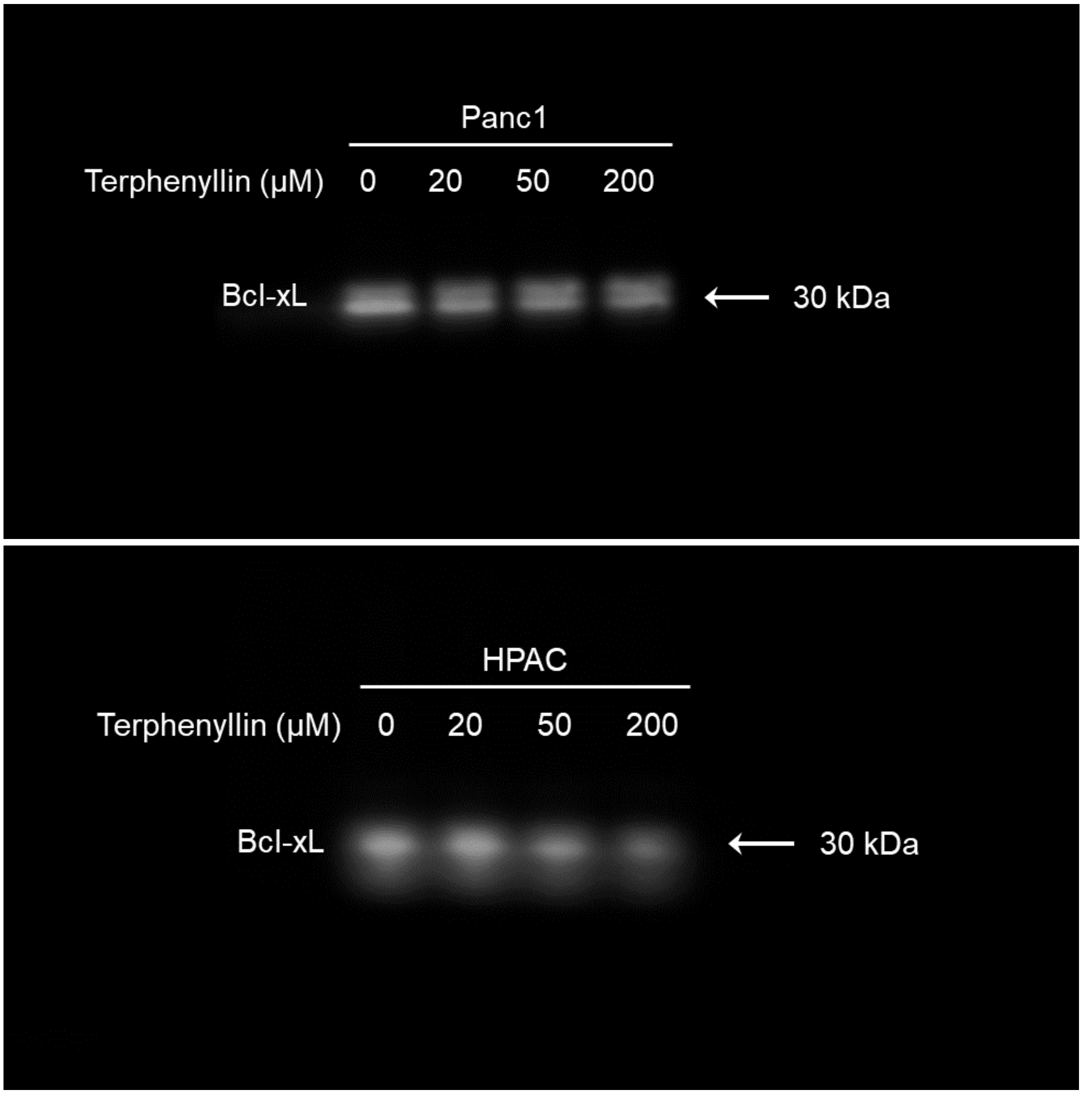

Supplementary Figure 8. Original images of anti-Caspase7 immunoblots in Figure 2C.

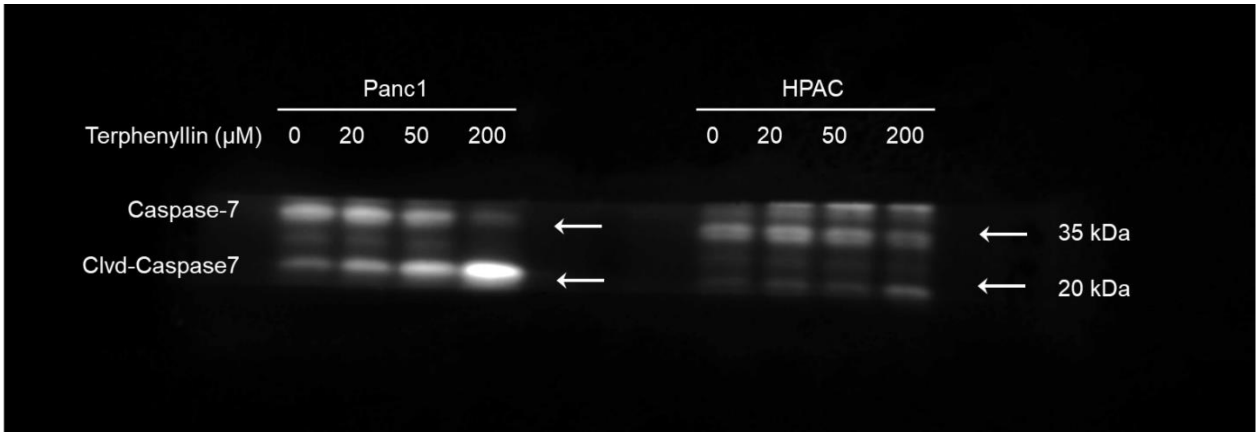

Supplementary Figure 9. Original images of anti-PARP immunoblots in Figure 2C.

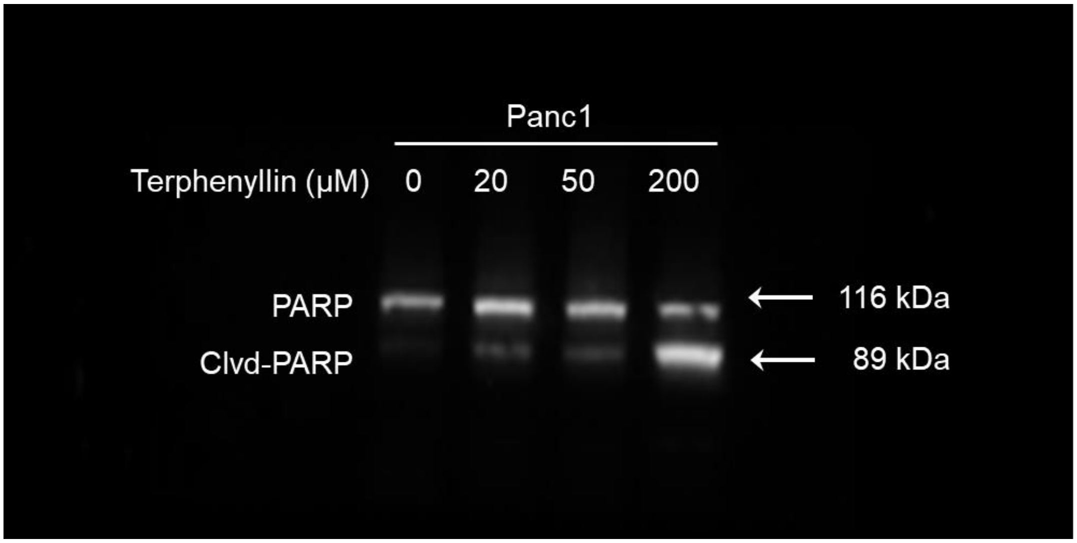

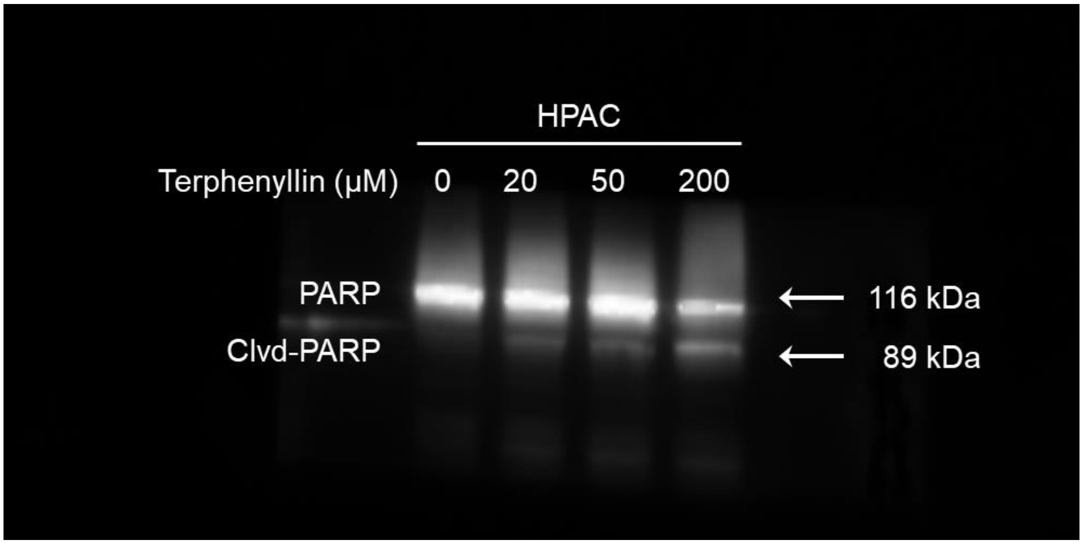

Supplementary Figure 10. Original images of anti-GAPDP immunoblots in Figure 2C.

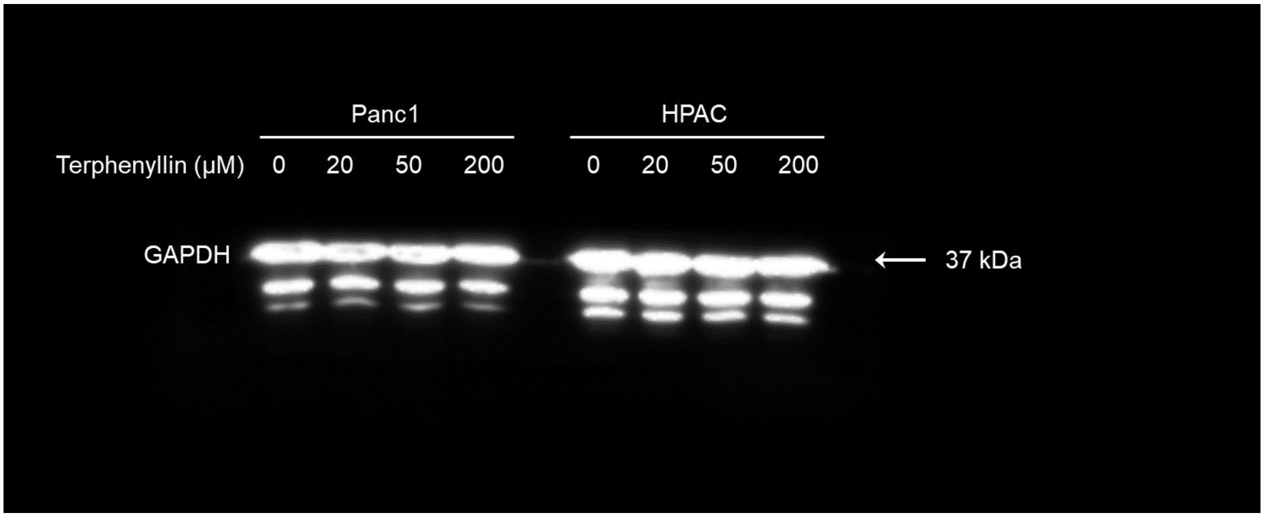

Supplement: Supplementary file 1 [file DataSheet_1.pdf]
